# Glucagon-Like Peptide-1 Receptor Imaging with [Lys^**40**^(Ahx-HYNIC-^**99m**^Tc/EDDA)NH_**2**_]-Exendin-4 for the Diagnosis of Recurrence or Dissemination of Medullary Thyroid Cancer: A Preliminary Report

**DOI:** 10.1155/2013/384508

**Published:** 2013-03-31

**Authors:** D. Pach, A. Sowa-Staszczak, A. Jabrocka-Hybel, A. Stefańska, M. Tomaszuk, R. Mikołajczak, B. Janota, M. Trofimiuk-Müldner, E. Przybylik-Mazurek, A. Hubalewska-Dydejczyk

**Affiliations:** ^1^Department of Endocrinology, Jagiellonian University Medical College, Kopernika 17, 31-501 Krakow, Poland; ^2^Radioisotope Center POLATOM, National Centre for Nuclear Research, 05-400 Otwock, Poland

## Abstract

*Introduction*. Epidemiological studies on medullary thyroid cancer (MTC) have shown that neither a change in stage at diagnosis nor improvement in survival has occurred during the past 30 years. In patients with detectable serum calcitonin and no clinically apparent disease, a careful search for local recurrence, and nodal or distant metastases, should be performed. Conventional imaging modalities will not show any disease until basal serum calcitonin is at least 150 pg/mL. The objective of the study was to present the first experience with labelled glucagon-like peptide-1 (GLP-1) analogue [Lys^40^(Ahx-HYNIC-^99m^Tc/EDDA)NH_2_]-exendin-4 in the visualisation of MTC in humans. *Material and Method*. Four patients aged 22–74 years (two with sporadic and two with MEN2 syndrome-related disseminated MTC) were enrolled in the study. In all patients, GLP-1 receptor imaging was performed. *Results*. High-quality images were obtained in all patients. All previously known MTC lesions have been confirmed in GLP-1 scintigraphy. Moreover, one additional liver lesion was detected in sporadic MTC male patient. *Conclusions*. GLP-1 receptor imaging with [Lys^40^(Ahx-HYNIC-^99m^Tc/EDDA)NH_2_]-exendin-4 is able to detect MTC lesions. GLP-1 scintigraphy can serve as a confirmatory test in MTC patients, in whom other imaging procedures are inconsistent.

## 1. Introduction

Medullary thyroid cancer (MTC) is a neuroendocrine neoplasm arising from the parafollicular cells, or C cells, of the thyroid. It accounts for nearly 5–10 % of thyroid malignancies. In nearly all MTC cases, cancer cells secrete calcitonin, a specific and highly sensitive biomarker—its measurement plays an important role in diagnosis and postoperative followup of patients [1–3]. The majority of MTCs are sporadic, but up to 25% of all cases result from a germ-line activating mutation of the *RET* protooncogene [4, 5]. Hereditary MTCs occur in the setting of the multiple endocrine neoplasia (MEN) syndrome type 2 (2A or 2B) or as familial MTC (FMTC)—a variant of MEN2A syndrome. The most common form of hereditary MTC is MEN 2A (approximately 80–90% of patients with hereditary MTC). Overall, the prognosis for patients with MTC is good. The 10-year survival rate is 75–85%. Approximately half of the MTC patients present with disease limited to the thyroid gland with a 10-year survival rate of 95.6%. One-third of patients present with locally invasive tumour or clinically apparent spread to the regional lymph nodes. Patients with regional disease have a 5-year overall survival rate of 75.5%. Recurrent disease develops in approximately 50% of patients with MTC [1, 6]. Neck ultrasound should be performed as a part of the initial evaluation of each patient with newly diagnosed MTC. Fine-needle aspiration (FNA) cannot always distinguish MTC based on the appearance of tumor cells alone, so the diagnosis is typically confirmed by immunostaining or by the measurement of calcitonin level in the washout fluid from FNA. This latter technique appears to be even more sensitive than cytology with immunohistochemistry [1]. 

The primary treatment for MTC is surgical resection. Total thyroidectomy with complete resection of central neck, paratracheal, and upper mediastinal lymph nodes is frequently needed. Currently, surgical excision is the only effective treatment for MTC. Patients who have clinically apparent disease are best treated with a minimum of total thyroidectomy and bilateral central neck dissection [7, 8]. Followup should start 2-3 months postoperatively by obtaining new baseline calcitonin levels. An undetectable basal serum calcitonin level is a strong predictor of complete remission. Patients with biochemical remission after initial treatment have only a 3% risk of recurrence during long-term followup [1, 2]. 

Calcitonin and stimulated calcitonin levels are very sensitive ways for detecting either residual or recurrent disease. When the postoperative calcitonin level is elevated, a careful search for metastases has to be performed prior to surgical exploration. Imaging techniques will not show any disease until basal serum calcitonin level exceeds 150 pg/mL. In patients with serum calcitonin lower than 150 pg/mL, localization of the disease should be focused on careful examination using neck ultrasound because such calcitonin levels are usually associated with locoregional disease. The optimal timing of this followup should be based on calcitonin and CEA (carcinoembryonic antigen) doubling times (DT), which are strongly correlated with disease progression [9–12]. 

There are some MTC patients in whom, despite of the elevated postoperative calcitonin levels and/or abnormal results of the pentagastrin test, there is no evidence of the disease in conventional imaging techniques. Prolonged delay in disease localization usually results in treatment failure even if the tumor recurrence/residue is finally detected. Molecular imaging techniques, based on the development of tracers which are taken up by MTC cells or are bound to MTC-specific receptors, could be applied in such group of patients. Therefore, besides the use of those well-known and commonly used radiotracers, such as labelled somatostatin analogues or mIBG, there are still clinical trials performed to find more specific and sensitive substances. Glucagon-like peptide 1 (GLP-1) labelled analogues have been considered as a promising tool for visualization of MTC. Physiologically GLP-1 (glucagon-like peptide-1) receptors have been found in organs like pancreas, blood vessels, stomach, or parafollicular C cells. Their expression is also observed in different types of neoplasms including MTC [13]. Both ^111^In-labeled GLP-1 analogue ([Lys^40^(Ahx-DTPA-^111^In)NH_2_]-exendin-4 and ^68^Ga/^99m^Tc labeled GLP-1 analogue exendin-4 were successfully used in patients with insulinoma [14–16]. ^99m^Tc labelled GLP-1 analogue, may improve the quality of images and radiation safety for patients and the staff due to many procedural advantages related to the isotope physical properties.

The question of the management of patients with local recurrence and contraindications to surgical intervention or patients with dissemination of the disease has not been solved. Those patients are left with few therapeutic choices. Chemotherapy is of limited value. [17]. External beam radiation therapy (EBRT) may be used only to control local disease [7, 8]. Serum calcitonin and CEA concentrations do not normalize after EBRT, but long-term stabilization may be achieved. Patients with metastatic disease can have debilitating symptoms from calcitonin excess and therefore may benefit from medical treatment with somatostatin analogues. Since MTC cells express somatostatin receptors, a radionuclide-targeted therapy with labelled octreotide and its derivates is another therapeutic option [17, 18]. 

Molecular-targeted therapy is yet another therapeutic strategy in MTC. With the discovery of the *ret *protooncogene and its integral role in the pathogenesis of MTC, a new class of therapeutics—tyrosine kinase inhibitors—has been developed [17, 19, 20]. It is necessary to develop other alternative therapeutic strategies to control tumour growth, possibly through interference with various cellular signalling pathways [17, 19, 20]. 

The aim of the paper is to present the first experience of our department with the new radiopharmaceutical [Lys^40^(Ahx-HYNIC-^99m^Tc/EDDA)NH_2_]-exendin-4 as a diagnostic tool in patients with suspected or confirmed recurrence or dissemination of MTC and to compare its performance with conventional imaging methods.

## 2. Material 

Four patients (1 female, 3 males, aged 22–74 years) were enrolled in the study. In all of them, recurrence or dissemination of MTC was suspected, based on previous imaging results and elevated calcitonin levels. In all patients, neck ultrasound was performed with fine-needle aspiration biopsy of suspected lesions in 3 cases. In two patients, neck and chest computed tomography (CT) and in one case abdominal CT were also performed. All subjects underwent somatostatin receptor scintigraphy (SRS).

*Patient 1 (J.S.).* Patient with sporadic MTC underwent total thyroidectomy with complete lymph nodes resection in the central neck and paratracheal compartment in 2004. In 2009, based on results of CT and SRS, patient was diagnosed with liver metastases and qualified to the peptide receptor radionuclide therapy (PRRT). Patient received 13.32 GBq (360 mCi) of  ^90^Y-DOTA-TATE. Treatment led to the stabilization of the disease. GLP-1 receptor imaging was performed to compare results with standard imaging procedures (US, CT and SRS). 

*Patient 2 (S.S.).* Patient with sporadic medullary cancer underwent total thyroidectomy with neck lymph nodes resection in 2003. In 2008, based on elevated calcitonin levels, neck ultrasound, fine-needle aspiration biopsy, neck and chest CT, and SRS dissemination to the neck and mediastinal lymph nodes were confirmed. Patient was disqualified from the surgery. Patient received 14.8 GBq (400 mCi) ^90^Y-DOTA-TATE in 2008, which resulted in the stabilization of the disease. GLP-1 receptor imaging was performed to compare results with conventional imaging methods (US, CT, and SRS).

This patient has also been diagnosed with chronic lymphocytic leukemia, diagnosed and operated for colon cancer in 2009, and metaplasia and dysplasia of the urinary bladder in 2010. In 2011 liver metastases from the colon cancer were diagnosed and patient was qualified to chemotherapy. 

*Patient 3 (K.G.).* Patient with MEN 2A syndrome underwent total thyroidectomy with neck lymph node resection in 2001. In 2009, abnormal pentagastrin test results were observed, but imaging studies (which ones) did not detect any lesions. In 2010, patient underwent bilateral adrenalectomy due to pheochromocytoma. In 2011, hypoechoic lesion on the left side of the neck u was revealed by ultrasound, but the biopsy was negative. Thyroid scintigraphy with ^99m^Tc and ^131^I were positive, but SRS was negative. The GLP-1 receptor imaging was ordered to facilitate discrimination between the thyroid remnant and MTC recurrence. 

*Patient 4 (Z.P.).* Patient with MEN 2B syndrome underwent total thyroidectomy with neck lymph node resection in 1990, followed by repeated surgery due to local recurrence in 1996. In 1993 patient underwent right adrenal and in 1997 left adrenal adrenalectomy due to pheochromocytoma. Based on chest CT, patient was diagnosed with lung metastases and local recurrence. SRS was negative. GLP-1 receptor imaging was ordered to confirm MTC recurrence in patient with discrepant results of other diagnostics images (positive CT, but negative SRS).

The detailed patient data are summarized in Table 1.

All patients gave their written informed consent to the local Medical College Ethics Committee which approved protocol. 

## 3. Methods

### 3.1. GLP-1 Analogue Scintigraphy

Patients were on a liquid diet for 1 day before the beginning of the examination and fasted on the day of the tracer injection. Each of them was carefully checked for any adverse reactions. Due to natural activity of GLP-1 (stimulation of insulin secretion), blood pressure and glucose values were monitored before and after injection of the compound at several time points.

### 3.2. Preparation of [Lys^40^(Ahx-HYNIC-^99m^Tc/EDDA)NH_2_]-Exendin-4

Technetium-99m labelled [Lys^40^(Ahx-HYNIC/EDDA)NH_2_]-exendin-4 was obtained from lyophilized kits prepared by the Institute of Atomic Energy, Radioisotope Center POLATOM. Exendin-4 (20 *μ*g) was modified C-terminally with Lys^40^-NH_2_, where the lysine side chain was conjugated with Ahx-HYNIC (Ahx is aminohexanoic acid). Tricine and EDDA as coligands for ^99m^Tc were added. The radiopharmaceutical preparation was carried out in the Nuclear Medicine Unit of the Endocrinology Department, Cracow University Hospital and was performed under aseptic conditions. Two-vial freeze-dried kits were used for radiolabelling with 0.3–1.5 mL pf ^99^Mo/^99m^Tc generator eluate (0.37–1.85 GBq) followed by 20 min incubation at 80°C. The TLC (thin layer chromatography) method was used for assessing the radiochemical purity of the compound. The mean injected activity was 740 MBq.

### 3.3. Imaging Technique

GLP-1 receptor imaging with the use of Lys^40^(Ahx-HYNIC-^99m^Tc/EDDA)NH_2_]-exendin-4 was performed at the Nuclear Medicine Unit of the Endocrinology Department in the University Hospital in Cracow. At the beginning, images were acquired with a dual-head, large field of view E.CAM gamma camera with low-energy high-resolution (LEHR) collimators (Siemens Healthcare, 2000). From 2010 all examinations were performed with the use of Symbia TruePoint T16 hybrid system also with LEHR collimators (Siemens Healthcare, 2007).

SPECT studies were performed at 2 time points, between 3-4 h and 5-6 h after the injection of the tracer. The SPECT examinations were done with 128 × 128 matrix, 64 images, 30 sec per image, step and shoot mode, noncircular orbit and dual-energy window for scatter correction. The acquired data were reconstructed using OSEM Flash 3D iterative reconstruction method with 8 subsets and 10 iterations. After the installation of the new hybrid device in the unit, SPECT/CT studies were carried out in all next patients with the same settings for the SPECT part of the study. 

In all cases low-dose CT imaging was performed for the attenuation correction and a improved localization of pathological uptake of the tracer.

The obtained images were assessed by the experienced nuclear medicine specialist.

## 4. Results

The average radiochemical purity of the administered compound, prepared according to manufacturer's instruction and determined by TLC, was higher than 90%.

The quality of obtained Lys^40^(Ahx-HYNIC-^99m^Tc/EDDA)NH_2_]-exendin-4 images was assessed by the nuclear medicine physician as very good.

In all patients results of scintigraphy with [Lys^40^(Ahx-HYNIC-^99m^Tc/EDDA)NH_2_]-exendin-4 corresponded to the results of previously performed imaging examinations. 

**Figure 1:**
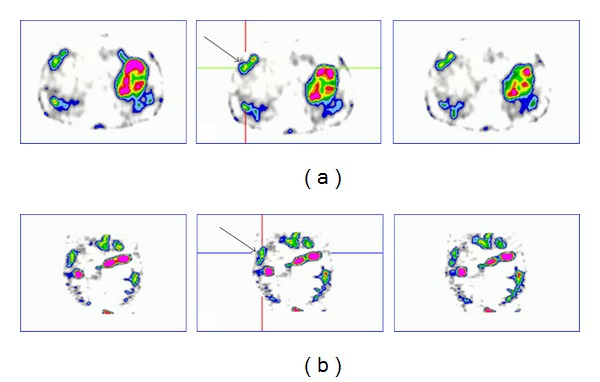
The positive results of GLP-1 receptor imaging in a 74-year-old patient (J.S.) with sporadic MTC; pathological uptake of the tracer in liver metastases is visualized. (a) Axial slices and (b) coronal slices.

In the first patient (J.S.) ^99m^Tc-GLP-1 receptor scintigraphy revealed in homogenous liver uptake and focally increased tracer uptake at the location of previously confirmed liver metastases (Figure 1). Moreover, an additional liver lesion not seen on SRS, was detected. Patient 1 is still available for followup with stable disease after PRRT. 

In the second patient (S.S.), ^99m^Tc-GLP-1 receptor scintigraphy performed after PRRT revealed small focal uptake in the neck. The image was comparable with SRS findings.

In the patient K.G. (patient 3), ^99m^Tc-GLP-1 receptor scintigraphy showed focal tracer uptake at the location of the ultrasonographically detected lesion on the left side of the neck. The patient was further qualified for the surgery.

In the patient Z.P. (patient 4), ^99m^Tc-GLP-1 receptor scintigraphy revealed tracer uptake at the location of the neck and chest lesions seen on CT. However, patient was disqualified from surgery due to heart failure.

No adverse reactions were observed after tracer injection. 

## 5. Discussion

MTC is still one of the most challenging endocrine cancers for both physicians and patients. In some MTC patients, despite of the elevated postoperative calcitonin levels and/or abnormal results of the pentagastrin test, there is no evidence of the disease in standard imaging procedures. Therefore searching for new targets for radioisotope diagnostics is warranted. ^99m^Tc(V)-dimercaptosuccunic acid (DMSA) was considered by many authors the agent of choice in the postoperative work-up of MTC. Sensitivities ranging from 50% up to 85% have been reported in patients with primary and recurrent MTC using planar scans. SPECT has increased the sensitivity of lesion detection. Shahram reported that ^99m^Tc(V)-DMSA had 91% sensitivity and 75% specificity for the detection of lung MTC compared to serum calcitonin as gold standard [21]. Another diagnostic modality is scintigraphy with ^99m^Tc-MIBI. Overall sensitivity and specificity of this agent range from 36% to 89% and 89% to 100%, respectively [22]. Uğur et al. have compared the sensitivity of ^99m^Tc-MIBI, ^201^Tl, and ^99m^Tc(V)-DMSA and shown them to be 47%, 19%, and 95%, respectively [23]. MIBG labelled with ^123^I or ^131^I, in spite of its high specificity (>95%), is of little clinical utility with a reported sensitivity of 30% [24]. Results from imaging with monoclonal antibodies including ^123^I-, ^131^I-, and ^111^In-labelled CEA, both whole antibody and fragments, and ^111^In-anticalcitionin antibody varied, ranging from 0% for anticalcitionin antibody to 78% for ^131^I-anti-CEA antibody [25]. Results of somatostatin receptor scintigraphy (SRS) using an octreopeptides labeled with either ^111^In-DTPA or ^99m^Tc-EDDA/HYNIC in MTC patients reported in the literature have been also extremely variable. The overall sensitivity of ^111^In-pentetreotide scintigraphy for the detection of MTC varies between 35 and 70% in different studies. Krenning et al. reported sensitivity of 65% in detecting MTC lesions by octreoscan, although the sensitivity was lower for liver metastases as a result of nonspecific hepatic uptake [26]. According to other authors, scintigraphy with ^111^In-DTPA-octreotide has shown a sensitivity of 50–75%, that is higher than radiolabelled MIBG [27] and similar or slightly superior to ^99m^Tc(V)-DMSA [28]. ^18^FDG-PET was more sensitive especially in detecting cervical, supraclavicular, and mediastinal lymph nodes, but failed to detect small lesions in the lungs and liver [29]. However, other studies have shown a lower sensitivity of ^18^FDG-PET when compared with CT [30]. Data from the study by Ong and coworkers suggested that ^18^FDG-PET is useful mainly in patients with calcitonin levels exceeding 1000 pg/mL (78% sensitivity), whereas it has limited value in patients with calcitonin levels below 500 pg/mL (20% sensitivity) [31]. Preliminary data suggest that ^18^F-L-dihydroxyphenylalanine (L-DOPA) PET may provide a better lesion detection than ^18^F-FDG for MTC lesions. Beheshti et al. observed that ^18^F-DOPA correctly visualized 81% of MTC lesions compared to 58% detected with ^18^F-FDG [32]. Hoegerle et al. reported an overall sensitivity of 63% for ^18^F-DOPA PET in 11 patients with MTC, which was lower than that observed with CT/MRI (it should be stressed that authors used a stand-alone PET system and not a hybrid PET/CT system), but higher than those observed with ^18^F-FDG and ^111^In-DTPA-octreotide scan [33]. 

Above-mentioned diversity of sensitivity and specificity of different imaging modalities used in patients with suspicion of recurrence or dissemination of MTC stresses the necessity of searching for new more accurate diagnostics tools. 

To the knowledge of the authors, this paper presents the first clinical experience with Lys^40^(Ahx-HYNIC-^99m^Tc/EDDA)NH_2_]-exendin-4 in the detection of the recurrence or dissemination of MTC. The quality of obtained images was high; however, the image fusion was mandatory for proper diagnosis in all reported cases. 

So far, the knowledge on GLP-1 application in MTC has emerged from experimental studies. The GLP-1 receptor protein expression was qualitatively and quantitatively investigated in many nonneoplastic and neoplastic human tissues with autoradiography method by Körner et al. [34]. They found GLP-1 receptor expression in 28% of medullary thyroid carcinomas examined and in 6% of normal human thyroid glands. GLP-1 receptor density of MTC cells was equal to 1,326 ± 264 dpm/mg tissue of receptor-positive cases. According to authors, medullary thyroid carcinomas exhibited a notable, but lower, GLP-1 receptor expression compared with, for example, pheochromocytomas. In recent paper by Gier et al. thyroids obtained from 12 individuals were examined for GLP-1 receptor protein by immunostaining [35]. GLP-1 receptor immunoreactivity was detected in approximately 10–30% of the tumor cells in five of the MTC cases. However, there was clear heterogeneity, with many calcitonin immunoreactive C cells being negative for GLP-1 receptor. The authors stated that these studies are in agreement with the work of Körner et al. [34].

Taking into account the GLP-1 receptor incidence and density in MTC, it seems that GLP-1 receptor imaging should not be used as the first-line diagnostic procedure in this group of patients. Nevertheless in case of patients with unclear or negative results of other imaging methods, but with clinical symptoms of MTC recurrence and/or elevated calcitonin concentration, this method gives the opportunity of localization of cancer tissue. Indeed, in our group of patients, the GLP-1 receptor imaging was carried out in two cases because of the discrepancy between results of performed diagnostic tests and resulted in confirmation MTC spread.

To sum up the GLP-1 receptor-expressing tumors, among others also MTC, are prospective candidates for in vivo targeting with Lys^40^(Ahx-HYNIC-^99m^Tc/EDDA)NH_2_]-exendin-4.

## 6. Conclusions

Scintigraphy with Lys^40^(Ahx-HYNIC-^99m^Tc/EDDA)NH_2_]-exendin-4 is able to detect the MTC lesions. It offers a new diagnostic tool to assess recurrence and staging of the disease in patients with MTC. GLP-1 receptor imaging should be considered as an alternative choice by clinicians especially in case of MTC patients in whom standard imaging techniques fail. However, further studies on the subject are needed.

## Figures and Tables

**Table 1:** Patients clinical data.

Initial	Age	Sex	Diagnosis	Genetic	CT	SRS	Diff. studies	GLP-1
J.S.	74	M	Dissem	Sporadic	+	+	US−	+
S.S.	70	M	Dissem	Sporadic	+	+	US+	+
K.G.	22	M	Recurr	MEN 2A	na	−	US+	+
Z.P.	60	F	Dissem	MEN 2B	+	−	US+	+

*F/M: female/male, −: negative result, +: positive result, na: not available, recur: recurrence, and dissem: dissemination.
